# Activity of D1/2 Receptor Expressing Neurons in the Nucleus Accumbens Regulates Running, Locomotion, and Food Intake

**DOI:** 10.3389/fnbeh.2016.00066

**Published:** 2016-04-12

**Authors:** Xianglong Zhu, David Ottenheimer, Ralph J. DiLeone

**Affiliations:** Department of Psychiatry, Yale University School of MedicineNew Haven, CT, USA

**Keywords:** nucleus accumbens, medium spiny neurons, designer receptors exclusively activated by designer drugs (DREADD), running, locomotion, food intake, energy expenditure, exercise

## Abstract

While weight gain is clearly promoted by excessive energy intake and reduced expenditure, the underlying neural mechanisms of energy balance remain unclear. The nucleus accumbens (NAc) is one brain region that has received attention for its role in the regulation of energy balance; its D1 and D2 receptor containing neurons have distinct functions in regulating reward behavior and require further examination. The goal of the present study is to investigate how activation and inhibition of D1 and D2 neurons in the NAc influences behaviors related to energy intake and expenditure. Specific manipulation of D1 vs. D2 neurons was done in both low expenditure and high expenditure (wheel running) conditions to assess behavioral effects in these different states. Direct control of neural activity was achieved using a designer receptors exclusively activated by designer drugs (DREADD) strategy. Activation of NAc D1 neurons increased food intake, wheel running and locomotor activity. In contrast, activation of D2 neurons in the NAc reduced running and locomotion while D2 neuron inhibition had opposite effects. These results highlight the importance of considering both intake and expenditure in the analysis of D1 and D2 neuronal manipulations. Moreover, the behavioral outcomes from NAc D1 neuronal manipulations depend upon the activity state of the animals (wheel running vs. non-running). The data support and complement the hypothesis of specific NAc dopamine pathways facilitating energy expenditure and suggest a potential strategy for human weight control.

## Introduction

Obesity is a complex disease that burdens individuals and health care systems. In a simplified view, energy intake and expenditure need to be balanced to maintain a normal body weight. Excess intake of high calorie food and reduced activity due to sedentary lifestyle have contributed to the increase in obesity rates (Prentice and Jebb, 2004; Garland et al., 2011). While controlling food intake and engaging in vigorous exercise is a clear strategy for weight loss, it is very difficult to achieve and maintain (Finlayson et al., 2011; Swift et al., 2014). A better description of neural mechanisms controlling intake and activity, and how these are integrated, is needed.

The dopamine system, especially the nucleus accumbens (NAc), is a potential neural substrate that can control both sides of the energy balance equation. In terms of energy intake, the NAc is known to regulate feeding. For example, food intake can be induced by blockade of specific glutamate receptors (Maldonado-Irizarry et al., 1995) or activation of mu opioid receptor (MOR; Zhang et al., 1998) in the NAc. In addition, local infusion of psycho-stimulant drugs, such as dopamine (Campbell et al., 1997), cocaine (Delfs et al., 1990), and amphetamine (Essman et al., 1993), into the NAc has long been known to increase locomotor activity. These data have led to models where that the NAc has a role in energy homeostasis by integrating intake and expenditure to adapt to environmental conditions (Beeler et al., 2012; Salamone and Correa, 2012).

How energy intake and activity-mediated expenditure are regulated and coordinated by neurons in the NAc remains unclear. Most neurons in the NAc are medium spiny neurons (MSNs), which can be divided into two major categories: dopamine receptor 1-like containing neurons (D1 neurons) and dopamine receptor 2-like containing neurons (D2 neurons; Albin et al., 1989; DeLong, 1990; Gerfen et al., 1990). D1 and D2 neurons in the NAc are physically intermingled and morphologically indistinguishable. Extensive studies using pharmacology have yielded inconsistent data on the role of D1 and D2 NAc neurons in food intake, and few studies have evaluated both activity and intake after NAc D1 and D2 manipulations (Baldo et al., 2002).

In this study, we assessed the effects of activation and inhibition of NAc D1 and D2 neurons on energy intake and activity-based expenditure. We used wheel running in addition to a locomotor activity assay to determine effects of D1 and D2 neuron manipulations on different types of activity. Notably, wheel running is rewarding and reinforcing for rodents, including wild strains (Sherwin, 1998; Meijer and Robbers, 2014) and has been proposed to parallel human voluntary exercise (Eikelboom, 1999; Rezende et al., 2009; Kelly et al., 2010; Garland et al., 2011). By measuring activity and intake in both running and non-running conditions, we are able to evaluate the role of D1 and D2 neurons on these different expenditure behaviors.

To achieve direct neuronal control and specificity, we employed D1 and D2-Cre mice together with designer receptors exclusively activated by designer drugs (DREADD) technology (Armbruster et al., 2007). DREADDs are genetically modified from the G-protein-coupled muscarinic receptor such that they are solely activated by clozapine-N-oxide (CNO), an otherwise pharmacologically inert compound, with high potency and efficacy. By locally injecting adeno-associated virus (AAV, serotype 2) expressing Gq (hM3Dq) or Gi (hM4Di)-coupled Cre-inducible DREADD into the NAc of D1 or D2-Cre mice, we achieved specific control (activation or inhibition) of each population of neurons and evaluated behavioral consequences (Alexander et al., 2009).

## Materials and Methods

### Subjects

D1-Cre (strain EY262, Gensat) and D2-Cre (strain ER44, Gensat) mice were maintained on a C57BL/6J background. Experiments were started when animals were 10–33 weeks of age. Within each group the maximum age difference was less than 4 weeks and the total animals used for analysis were: D1-Cre Gq *n* = 8, D1-Cre Gi *n* = 8, D2-Cre Gq *n* = 5, and D2-Cre Gi *n* = 14. We did not detect significant behavioral differences between males (*n* = 23) and females (*n* = 12) in the study, and data from both sexes were used. All procedures were approved by the Yale Institutional Animal Care and Use Committee.

### Virus Expression

We used commercially available AAV-hSyn-DIO-hM3D(Gq)-mCherry and AAV-hSyn-DIO-hM4D(Gi)-mCherry, obtained from UNC Gene Therapy Center Vector Core.

### Stereotaxic Surgery

A mixture of ketamine (100 mg/kg body weight) and xylazine (10 mg/kg body weight) delivered via intraperitoneal (i.p.) injection was used for anesthesia. Animals were placed in a stereotaxic frame (Stoelting, Wood Dale, IL, USA). After craniotomy, viruses were injected into the NAc bilaterally (0.5 μl/side) through a 32 gauge syringe over 5 min at the following coordinates (all values given relative to bregma): +1.4 mm rostral, ±0.7 mm lateral, −4.5 mm ventral. Mice recovered for 2–3 weeks to allow for optimal virus expression before the initiation of behavioral studies.

### Wheel Running

**Figure 1:**
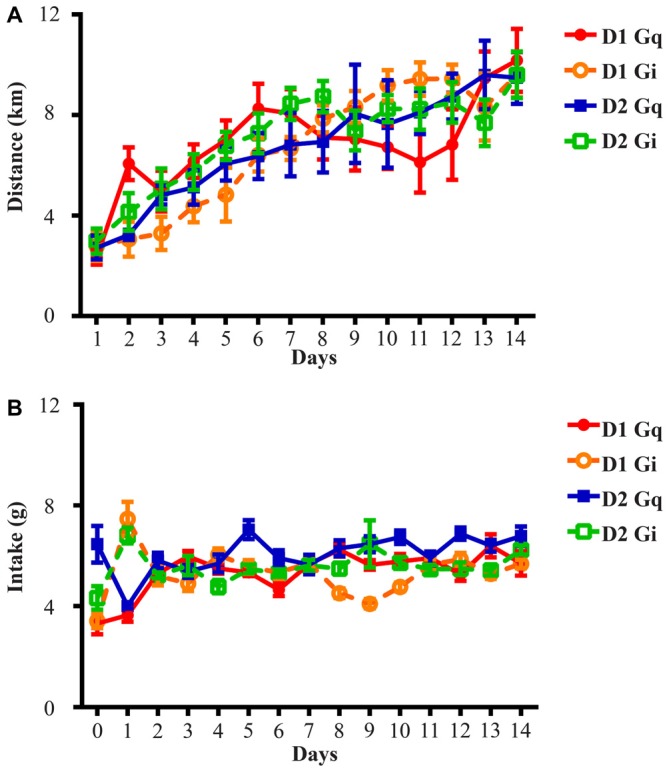
**Running distance and food intake increase and stabilize during the 14-day exposure to running wheels. (A)** All four groups of animals in this study rapidly adapted to wheel running and average daily running distance was 2.71 ± 0.14 km on Day 1 and 9.72 ± 0.16 km on Day 14. **(B)** Average daily food intake across four groups of animals was 4.38 ± 0.73 g on Day 0 (the day before onset of wheel access), 5.48 ± 0.97 g on Day 1 and 6.10 ± 0.26 g on Day 14. D1 Gq stands for the cohort of D1-Cre mice with Gq-coupled DREADD expression in the NAc (*n* = 7). Similar abbreviations are used for D1 Gi (*n* = 6), D2 Gq (*n* = 5), and D2 Gi (*n* = 14).

**Figure 2:**
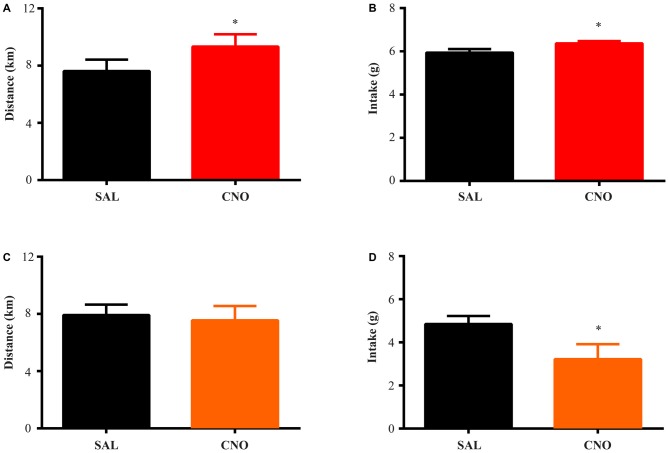
**Activation of D1 neurons in the nucleus accumbens (NAc) increases food intake and running distance, while inhibition of D1 neurons suppresses food intake without effecting running distance.** D1-Cre mice with Cre-inducible Gq-DREADD expression in the NAc were given access to wheels for 2 weeks of training. Saline or clozapine-N-oxide (CNO) was injected i.p. 2 h before the onset of the dark cycle on two consecutive days, and running distance and food intake over 24 h after injection were assessed. **(A)** Activation of D1 neurons in the NAc increased running distance (paired *t* test *P = 0.0007, *n* = 7) and food intake (paired *t* test *P = 0.0008, *n* = 7) during 24 h after injection **(B)**. **(C)** Inhibition of D1 neurons in the NAc did not change running distance but decreased food intake **(D)** over 24 h (paired *t* test *P = 0.0026, *n* = 8).

**Figure 3:**
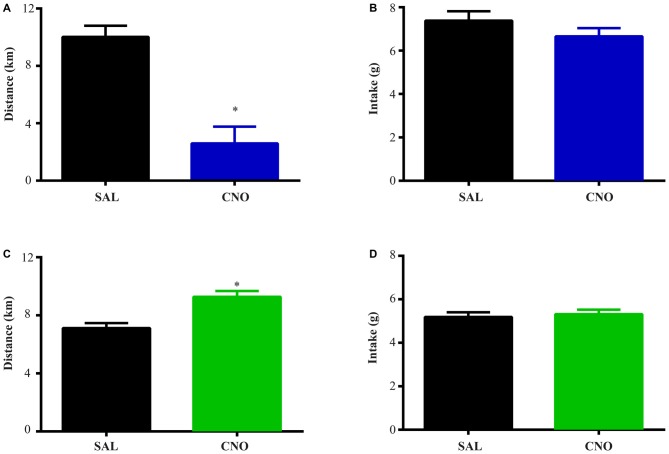
**Activation of D2 neurons in the NAc suppresses running without affecting food intake, while inhibition of D2 neurons increases running without effects on intake.** The same experimental procedures in Figure 2 were performed on D2-Cre mice with Cre-inducible Gq/Gi-DREADD expression in the NAc. **(A)** 24 h running distance was suppressed by activation of D2 neurons in the NAc (paired *t* test *P = 0.0054, *n* = 4) while food intake was not affected **(B). (C)** 24 h running distance was increased by inhibition of D2 neurons in the NAc (paired *t* test *P = 0.0014, *n* = 14) with no effects on food intake **(D)**.

The mice were maintained on a 12 h light/dark cycle, with lights on at 7 A.M. and lights off at 7 P.M., and had *ad libitum* access to water, food, and running wheels with a diameter of 11 cm, corresponding to 0.3454m per revolution. At least 14 days of voluntary wheel running were allowed in order to stabilize running and intake before the test period. Rotation counts were recorded by “clocklab” software (Actimetrics, Wilmette, IL, USA). Saline was given i.p. (0.1 ml/10 g body weight) first and 10 mg/kg CNO was injected the following day. Injections were performed 2 h prior to onset of dark cycle. These procedures were completed three times with 2–3 days between repeats, and data collected from saline and CNO days were averaged for within-subject comparison. Due to equipment malfunction, two mice from the D1-Cre Gi cohort were excluded from the pre-treatment wheel running and the concurrent intake analysis (Figure 1). Additionally, one mouse from the D1-Cre Gq, and one mouse from the D2-Cre Gq cohort were excluded from the wheel running and the concurrent intake analysis (Figures 2A,B, 3A,B).

### Locomotor Activity Assay

**Figure 4:**
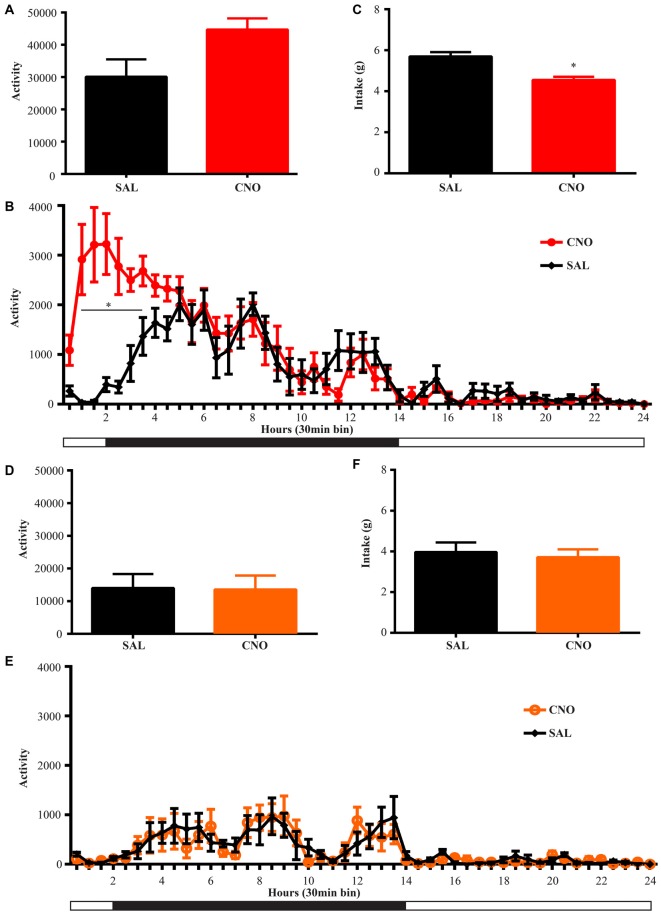
**Activation of D1 neurons in the NAc decreases intake accompanying a mild increase in locomotion, while inhibition of D1 neurons in the NAc has no effects on locomotion and intake. (A)** CNO activation of Gq-DREADD in the D1 neurons in the NAc tended to induce locomotion (paired *t* test *P* = 0.06, *n* = 7) over 24 h after injection and locomotor elevation **(B)** was robust during 0.5~3.5 h after injection (*F*_(47,564)_ = 5.877, *P < 0.0001, Bonferroni’s *post hoc*: *P < 0.05 over bin 2~7, saline baseline vs. CNO treatment). **(C)** Food intake was decreased during 24 h after injection (paired *t* test *P = 0.0007, *n* = 7). **(D,E)** CNO activation of Gi-DREADD in the D1 neurons in the NAc (*n* = 7) did not change locomotor activity over 24 h, and did not affect food intake **(F)**. Black bars represent dark period and white bars represent light period.

**Figure 5:**
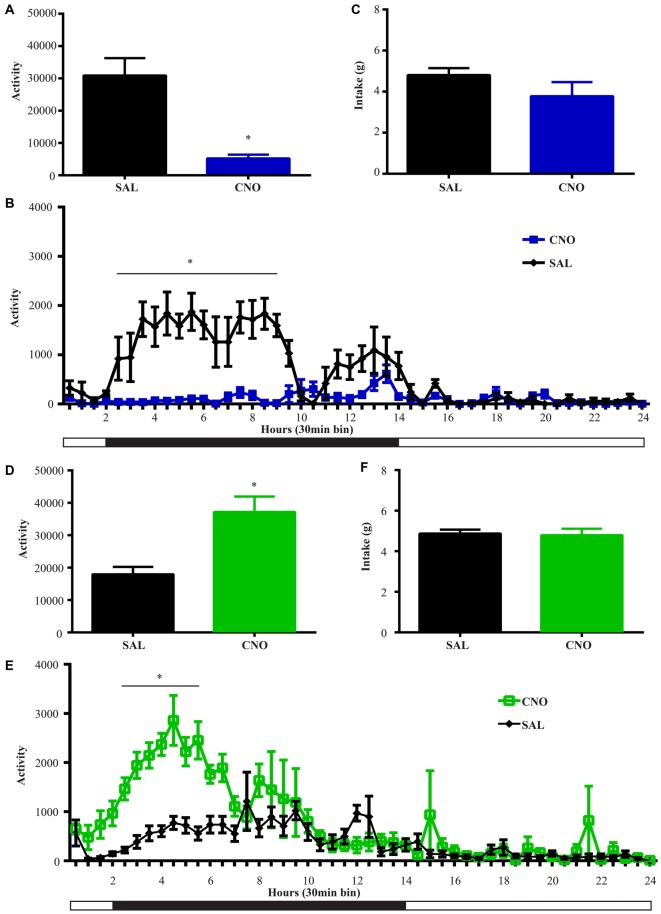
**Activation of D2 neurons in the NAc suppresses locomotion without affecting food intake, while inhibition of D2 neurons increases locomotion without effects on intake. (A)** CNO activation of Gq-DREADD in the D2 neurons in the NAc reduced total locomotor activity over 24 h (paired *t* test *P = 0.0064, *n* = 5), with the most robust effect during 2.5~9 h after injection (*F*_(47,376)_ = 7.474, *P < 0.0001, Bonferroni’s *post hoc*: *P < 0.05 over bin 5~18, saline baseline vs. CNO treatment) **(B). (C)** Food intake during 24 h after activation of D2 neurons was not affected. **(D)** CNO activation of Gi-DREADD in the D2 neurons in the NAc robustly induced locomotion over 24 h (paired *t* test *P = 0.0005, *n* = 11), with the most robust effect during 2.5~6.5 h after injection (*F*_(47,940)_ = 3.789, *P < 0.0001, Bonferroni’s *post hoc*: *P < 0.05 in bins 5–11) **(E). (F)** Food intake was not affected during 24 h after CNO injection. Black bars represent dark period and white bars represent light period.

All mice were habituated to the locomotor chambers (Med Associates, St. Albans, NY, USA) for 1 day prior to treatment with the same light/dark cycle as in wheel running period. Following habituation day, saline i.p. (0.1 ml/10 g body weight) injection was conducted 2 h prior to onset of dark cycle. 10 mg/kg CNO was injected at the same time the next day and locomotor activity was recorded in 30 min bins. Data from some animals were excluded from analysis of locomotion and corresponding intake due to technical artifacts (1 in D1-Cre Gq, 1 in D1-Cre Gi, 0 in D2-Cre Gq, 3 from D2-Cre Gi; Figures 4, 5).

### Statistical Procedures

GraphPad Prism 6.0 was used to calculate statistical tests. To analyze the within-subject difference between treatments (saline vs. CNO), all *t*-tests performed were two-tailed, paired. When applicable, two-way ANOVAs with repeated measurements were assessed and *post hoc* analyses were conducted. All error bars shown in the graphs represent ± SEM.

### Immunofluorescence for Viral Infection Assessment

D1/2-Cre C57BL/6J transgenic mice were deeply anesthetized and intra-cardially perfused using 10% formalin. Following 30% sucrose cryoprotection, the brain was sectioned at 40 μm thickness on a freezing microtome and stored in 1 × PBS with 0.01% sodium azide to prevent bacterial growth. Immunofluorescence was performed by pretreating sections in 2% Triton X-100/1 × PBS for 30 min before blocking buffer incubation for 60–90 min (3% donkey serum, 0.3% Triton X-100, 1 × PBS). Primary antibody (anti-DsRed, 1:500, Clontech, Palo Alto, CA, USA) was diluted accordingly in blocking buffer and sections were incubated overnight at room temperature. After 2 h incubation of secondary antibody (donkey anti-rabbit, Alexa Fluor 555, 1:500, Life Technologies, Carlsbad, CA, USA), tissue was visualized and images were captured with a fluorescent microscope (Zeiss, Thornwood, NY, USA) using standard TRITC filters.

## Results

### NAc D1 Activity Positively Regulates Food Intake During Wheel Running

The D1-Cre mice with Cre-inducible Gq or Gi-DREADD expression in the NAc were given access to wheels for at least 14 days before saline or CNO administration. For both D1-Cre Gq and Gi cohorts, total running distance increased during the entire period (Figure 1A) while food intake also increased and then stabilized after the first few days (Figure 1B). Saline or CNO was injected i.p. 2 h before onset of the dark cycle on respective days, and daily running distance and food intake were measured on three separate trials. When D1 neurons expressing Gq-DREADD in the NAc were activated with CNO administration, mice increased running distance (Figure 2A) over 24 h accompanying an increase in food intake during the same period (Figure 2B). In contrast, when NAc D1 neurons expressing Gi-DREADD were inhibited with CNO, the mice decreased food intake (Figure 2D) but running distance over 24 h after injection was not affected (Figure 2C). These data are consistent with a role for D1 neurons in both intake and expenditure (Baldo et al., 2002) in wheel running mice.

### D2 Neurons in the NAc Bi-Directionally Regulate Running Distance Without Affecting Food Intake

The same tests were performed on D2-Cre mice with Cre-inducible Gq/Gi-DREADD expression in the NAc. Here, the activity of D2 neurons in the NAc negatively correlates with running distance. When mice with Gq-DREADD in D2 neurons were treated with CNO, running distance over 24 h after injection was reduced (Figure 3A), whereas CNO treatment of D2-Cre, Gi-DREADD mice increased running distance (Figure 3C). In both cases, food intake during the same time was not affected (Figures 3B,D). These results suggest a more prominent role of NAc D2 neurons in activity rather than intake under wheel running conditions.

### Activation of D1 Neurons in the NAc Briefly Elevates Locomotion

Animals were habituated in locomotor test boxes (without wheels) for 1 day and then were given saline injection i.p. on the next day. On the third day, CNO was administered and D1-Cre animals with Gq-DREADD exhibited a near significant increase (*P* = 0.06) in 24 h locomotor activity (Figure 4A) with clear effects in the first 6 h (Figure 4B). Surprisingly, food intake during the same 24 h period was reduced (Figure 4C), and the reduction occurred in later hours within the test period (food intake 0–6 h after injection was 2.44 ± 0.11 g on saline day vs. 2.61 ± 0.31 g on CNO day, paired *t* test *P* = 0.62; food intake 6–24 h after injection was 3.36 ± 0.18 g on saline day vs. 2.07 ± 0.32 g on CNO day, paired *t* test *P = 0.0042). Conversely, when NAc D1 neurons were inhibited, neither food intake nor locomotor activity was affected (Figures 4D–F).

### NAc D2 Activity Level Inversely Regulates Locomotion Without Affecting Food Intake

We performed the same experiment to examine the effects of activation and inhibition of D2 neurons on locomotion and intake. Similar to the results from the wheel running condition, activation of D2 neurons with Gq-DREADD in the NAc suppressed total locomotion over 24 h after injection (Figure 5A) with the strongest effects between 3–9 h (Figure 5B). In contrast, inhibition of D2 neuron activity in the NAc with Gi-DREADD increased total locomotion over 24 h after injection (Figure 5D) with the strongest effects in the first 6 h (Figure 5E). No difference in food intake was found during the same 24 h periods (Figures 5C,F).

### Viral Expression Assessment

**Figure 6:**
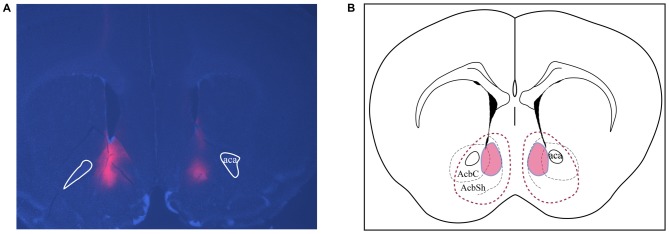
**Assessments of placements and viral expression in the NAc. (A)** Representative micrograph shows the expression and distribution of Cre-inducible DREADD virus with mCherry (red). DAPI (blue) depicts the brain coronal section containing the NAc. **(B)** Schematic drawing of the viral expression distribution across all four cohorts of animals. Red areas circled by solid lines indicates the common region with expression in each individual while the area circled by dashed lines shows the largest viral expression area among all animals used in this study. AcbSh and AcbC, shell and core regions of the NAc, respectively; Aca, anterior commissure.

All brains were perfused and sectioned and immunofluorescence was performed to confirm the placement of viral expression. Figure 6A is a representative image showing bilateral targeting in the NAc, in which the red area represents expression of mCherry-tagged Cre-inducible DREADD virus. Animals with bilateral hits as shown in Figure 6B (red outlines) were included for analysis. All animals had expression in the shell region and most also had expression in the core.

## Discussion

In the present study, we have demonstrated that D1 and D2 neurons in the NAc play different roles in modulating energy balance and that these effects vary depending on the environment. With wheel access, activity of D1 neurons in the NAc mainly controls food intake while NAc D2 neuron manipulations affect running distance. When wheels are not available, activation and inhibition of D2 neurons in the NAc still bi-directionally modulates locomotor activity without affecting intake while activation of D1 influences both locomotion and food intake.

The cellular (D1 vs. D2) and regional (NAc) specificity of the present study helps to complement and clarify some of the present literature on dopamine pathways. The canonical view of motor control in the dorsal striatum suggests that the neural activity in the direct pathway (D1 neurons) enhances movement, while neural activity in the indirect pathway (D2 neurons) inhibits movement (Albin et al., 1989; Gerfen et al., 1990). This view is supported by direct manipulations of these populations in the dorsal striatum (Kravitz et al., 2010). This opposing action is consistent with our finding here that activation of the ventral striatal D1 neurons promoted both running and general locomotion while activation of NAc D2 neurons inhibited running and locomotion. While direct manipulation of NAc neurons during running has not been previously reported, over-expression of deltaFosB within the direct pathway of the NAc and dorsal striatum increases running, whereas deltaFosB overexpression within the indirect pathway decreases running (Werme et al., 2002).

### Contrasting Effects of NAc D1 and D2 Neuron Activation in Regulating Energy Balance During Wheel Running

Studies using systemic administration of dopamine agonists or antagonists have produced conflicting results on the role of dopamine receptors in running. Rhodes et al., found that D1 and D2 antagonists reduced running distance in a strain of selective high runners (Rhodes et al., 2001; Rhodes and Garland, 2003) while Knab et al. (2012) showed that D1 agonist, but not D1 antagonist, reduced running distance in a different strain of high running mice. These studies highlight genetic variations that are of importance, but do not identify specific neural circuits mediating the effects of dopamine. Moreover, the effects of pharmacological reagents on neural activity are often complex. The present use of DREADD-expressing viruses allowed for specific manipulation of D1 and D2 neural activity within the NAc and demonstrates that NAc D1 neurons play a dominant role in controlling food intake while NAc D2 neurons primarily regulate wheel running distance. These effects help to identify likely neural mechanisms by which dopamine influences both intake and activity. For example, high NAc dopamine would increase food intake via D1 neuron activation while also increasing energy expenditure via D2 neuron inhibition. In contrast, when dopamine is low in the NAc, the present data suggest that lower D1 and higher D2 neuron activity would cause reduction in both energy intake and expenditure.

### Opposite Effect of NAc D1 and D2 Neurons on Locomotion

When wheels are not accessible, activity changes of both D1 and D2 neurons in the NAc affect general locomotion. Activation of NAc D1 neurons transiently increased locomotion, while D2 neuron activation decreased locomotion. These results are in agreement with the study by Baldo et al. (2002) where they found that D1 and D2 antagonist delivered into NAc could reduce ambulatory locomotion with more pronounced effect by the D1 antagonist. In the present study, D1 neuron activation reduced intake in non-running conditions while D1 neuron inhibition had no effect on intake. However, the subtle changes in meal patterns reported by Baldo et al. (2002) after D1 antagonist treatment might not be seen in our study design.

The effects of D1 and D2 stimulation in the non-running condition are consistent with the hypothesis of dopamine facilitating energy expenditure (Beeler et al., 2012; Salamone and Correa, 2012). As stated by Beeler et al. (2012), under conditions where the energy supply is plenty, elevated dopamine would promote exploration and energy expenditure. The present work demonstrates how D1 and D2 neurons would mediate these effects since activation of NAc D1 neurons and inhibition of NAc D2 neurons mainly elevated locomotion (energy expenditure) without increasing intake. In contrast, decreased dopamine, likely causing D1 neuron inhibition and D2 neuron disinhibition, was hypothesized to reduce locomotion and energy expenditure. The results here suggest that under non-running conditions, the influence of reduced dopamine on activity may be D2 neuron mediated. The reduction in food intake following D1 neuron activation (Figure 4C) is also consistent with higher expenditure-related behavior during conditions of higher dopamine. It is notable that this reduced intake occurred at a later time after CNO administration (6–24 h post-injection), suggesting a delayed response to D1 neuronal activation.

The notable baseline differences between Gq and Gi groups could be due to the viruses or other environmental variables (e.g., age differences, temperature, season). We acknowledge this and did not compare between groups but rather completed within-subject analysis to determine the effect of D1/2 neuronal activity changes.

### Activity Changes of NAc Neurons Result in Different Outcomes Under Different Environmental Contexts

As obesity and related diseases have become more prevalent in Western societies, increase in sedentary lifestyles, as well as dietary changes, have been identified as potential causes. In the present study, we employed two behavioral environments: one had wheel access with running distance measured for energy expenditure while the other was a standard home cage with expenditure assessed by locomotor activity. We were able to observe different behavioral outcomes under these distinct expenditure conditions. It should be noted that the energy expenditure condition used here, chronic running, might lead to specific physiological adaptations that are not seen under all “high expenditure” conditions (e.g., exploration of new space). For D1 neurons, inhibition decreases intake under high expenditure conditions with no effect in low expenditure conditions. This has implications for how we might view interventions to influence food intake, and potentially body weight, in people with different levels of expenditure. In contrast, the D2 neuron manipulations highlight this pathway as a potentially better target for activity control under both high and low expenditure conditions. That is, specific inhibition of NAc D2 neurons would increase the amount of daily activity or exercise without affecting food intake, which could aid weight control.

## Author Contributions

XZ designed experiments, conducted experiments, analyzed data, interpreted results, and drafted the manuscript. DO bred and genotyped transgenic mice, interpreted results, and edited the manuscript. RJD conceived of experiments, designed experiments, interpreted results, and revised the manuscript. All authors approved the submitted version of the manuscript.

## Conflict of Interest Statement

The authors declare that the research was conducted in the absence of any commercial or financial relationships that could be construed as a potential conflict of interest.
